# Evaluation of cardiolipin nanodisks as lipid replacement therapy for Barth syndrome

**DOI:** 10.7555/JBR.32.20170094

**Published:** 2017-12-17

**Authors:** Nikita Ikon, Fong-Fu Hsu, Jennifer Shearer, Trudy M. Forte, Robert O. Ryan

**Affiliations:** 1. Children's Hospital Oakland Research Institute, Oakland, CA 94609, USA; 2. Department of Medicine, School of Medicine, Washington University, St. Louis, MO 63110, USA; 3. Department of Biochemistry, University of Nevada, Reno, NV 89557, USA.

**Keywords:** mitochondria, cardiomyopathy, nanoparticles, drug delivery

## Abstract

Barth syndrome (BTHS) is a mitochondrial disorder characterized by cardiomyopathy and skeletal muscle weakness. Disease results from mutations in the tafazzin (*TAZ*) gene, encoding a phospholipid transacylase. Defective tafazzin activity results in an aberrant cardiolipin (CL) profile. The feasibility of restoring the intracellular CL profile was tested by *in vivo* administration of exogenous CL in nanodisk (ND) delivery particles. Ninety mg/kg CL (as ND) was administered to doxycycline-inducible *taz* shRNA knockdown (KD) mice once a week. After 10 weeks of CL-ND treatment, the mice were sacrificed and tissues harvested. Liquid chromatography-mass spectrometry of extracted lipids revealed that CL-ND administration failed to alter the CL profile of *taz* KD or WT mice. Thus, although CL-ND were previously shown to be an effective means of delivering CL to cultured cells, this effect does not extend to an *in vivo* setting. We conclude that CL-ND administration is not a suitable therapy option for BTHS.

## Introduction

Barth Syndrome (BTHS) is a rare, life threatening X-linked recessive disorder characterized by cardiomyopathy, skeletal muscle weakness, low weight gain, neutropenia, and 3-methylglutaconic aciduria^[1]^. The underlying cause of BTHS has been traced to mutations in the tafazzin (*TAZ*) gene^[2–3]^, which encodes a phospholipid transacylase, termed tafazzin^[4]^. Loss of tafazzin activity leads to a deficiency in cardiolipin (CL), an important phospholipid component of the mitochondrial inner membrane (IM). Additional changes include increased CL molecular species heterogeneity and accumulation of monolyso (ML) CL^[5]^. Given the key structural role CL plays in the IM of mitochondria^[6]^, it is not surprising that BTHS subjects manifest ultrastructural changes to this organelle^[7]^, as well as defective energy metabolism, particularly in cardiac tissue and skeletal muscle^[8–9]^. Normally, heart and skeletal muscle mitochondria are highly enriched in a single CL molecular species, tetralinoleoyl CL^[10]^. Establishment and maintenance of this molecular species composition is dependent upon acyl chain remodeling reactions that involve tafazzin transacylase activity^[11]^. When tafazzin is missing or defective, major changes in CL content and composition occur.

To date, treatment of BTHS is largely symptomatic and directed toward alleviating problems associated with cardiomyopathy, skeletal myopathy and neutropenia. However, earlier research suggests that compensating for defective tafazzin activity may be a feasible approach. For example, Valianpour *et al.*^[12]^ investigated whether linoleic acid supplementation of the growth medium for cultured BTHS fibroblasts would have an effect on tetralinoleoyl CL levels. These authors reported a time and dose dependent increase in tetralinoleoyl CL following supplementation with linoleic acid, suggesting that a deficiency in tafazzin-mediated CL remodeling can be bypassed by increasing substrate availability for direct *de novo* synthesis of tetralinoleoyl CL.

To investigate the underlying cause of neutropenia in BTHS, Makaryan *et al.*^[13]^ used HL60 myeloid progenitor cells as a model system. When these cells were transfected with a *TAZ*-specific short-hairpin RNA (shRNA), increased apoptosis was observed. Subsequently, in an attempt to bypass the deficiency, *TAZ* knockdown (KD) HL60 cells were incubated with CL nanodisks (ND), water soluble nanoscale particles formed upon incubation of an aqueous dispersion of tetralinoleoyl CL with an amphipathic apolipoprotein^[14–16]^. ND-mediated delivery of tetralinoleoyl CL to cultured *TAZ* KD HL60 cells delivered exogenous CL to mitochondria and attenuated the apoptotic response^[16]^.

Based on these *in vitro* results, it was hypothesized that *in vivo* administration of CL-ND may affect mitochondrial CL levels. A doxycycline (dox)-inducible *taz* shRNA KD mouse has been reported that recapitulates the BTHS phenotype. These mice exhibit an abnormal CL profile, mitochondrial structural abnormalities, impaired weight gain and adult-onset cardiomyopathy^[17–19]^. At eight weeks of age, Acehan *et al. *^[17]^ observed a dramatic decrease in CL content in cardiac and skeletal muscle as well as a shift toward saturated CL molecular species. There was also a substantial increase in levels of MLCL, resulting in a markedly elevated MLCL/CL ratio. Pronounced defects in both cardiac and skeletal muscle were noted at eight months, including extreme morphological changes in mitochondria and severe left ventricular dysfunction. Taken together, this murine model of BTHS recapitulates phenotypic features of the human disorder. Herein, these mice were employed in experiments designed to test the hypothesis that administration of CL- ND over a 10 week period normalizes the content and composition of CL in key tissues and, thereby, confers protection against manifestation of the BTHS disease phenotype.

## Materials and methods

### CL-ND formulation

Tetralinoleoyl CL [(18:2)4-CL] (5 mg, Avanti Polar Lipids Inc, Alabaster, AL, USA) was transferred to a glass tube and solvent evaporated under a stream of N2 gas, forming a thin film on the vessel wall. Residual solvent was removed under vacuum. The prepared lipid was dispersed in PBS (20 mmol/L sodium phosphate, 150 mmol/L sodium chloride, pH 7.0) followed by the addition of 2 mg recombinant murine apolipoprotein (apo) A-I^[20]^ in a final volume of 0.55 mL. Bath sonication of the turbid CL-apoA-I mixture induced sample clearance, an indication that CL-ND had formed^[16]^. CL-ND preparations were sterile-filtered (0.22 µm) prior to administration to mice.

### Animals

C57BL/6J *taz* shRNA KD mice and wild type (WT) littermates were obtained from The Jackson Laboratory. WT female and hemizygous male *tet*-on shRNA KD mice were used for breeding. Breeding females were maintained on dox (625 mg/kg in chow) from one week before mating until pups were weaned, except during breeding (to prevent dox-related infertility in breeding males). Upon weaning, pups were placed on dox for the remainder of the study.

### CL-ND toxicity testing

WT C57BL/6 mice were administered a single intraperitoneal (IP) injection of 0, 30, 60, 90 and 150 mg/kg CL in the form of CL-ND. After 24 hours, blood was collected by submandibular vein bleed, centrifuged at 8,000 *g* at 4ˆC for 10 minutes; plasma was recovered and stored at -80ˆC until use. Plasma alanine transaminase (ALT) and aspartate transaminase (AST), as well as creatinine and blood urea nitrogen (BUN), were analyzed by the UC Davis Comparative Pathology Laboratory.

### CL-ND injection studies

Only male mice were used in experiments and all were maintained on dox throughout the treatment period. CL-ND administration was by IP injection, starting when pups reached 4 weeks of age and continued for 10 weeks. At the conclusion of the treatment phase, mice were euthanized, tissues harvested and frozen (-80ˆC) until extracted. The animal protocol employed was approved by Children's Hospital Oakland Research Institute's Institutional Animal Care and Use Committee. Five groups of mice were injected as follows: Group 1: *taz* shRNA KD mice (*n* = 6) administered a weekly bolus injection of 90 mg/kg CL (as ND). Group 2: *taz* shRNA KD mice (*n* = 6) administered a daily injection (5 days per week) of 18 mg/kg CL (as ND). Group 3: *taz* shRNA KD mice (*n* = 5) administered a weekly injection of PBS (corresponding to the volume of the CL-ND injection). Group 4: WT mice (*n* = 6) administered a weekly bolus injection of 90 mg/kg CL (as ND). Group 5: WT mice (*n* = 6) administered a weekly injection of PBS (corresponding to the volume of the CL-ND injection). Mice were weighed weekly and monitored for outward signs of health over the course of the treatment period.

### Extraction of CL from tissues

Previously frozen heart, skeletal muscle, and liver tissues were homogenized using a FastPrep FP120 Cell Disruptor and Lysing Matrix M (MP Biomedicals, Santa Ana, CA, USA), according to the manufacturer's instructions. Homogenates were further disrupted by sonication and extracted as previously described^[16]^. Tetramyristoyl CL [(14:0)4-CL] was used as internal standard (heart= 10 µg, muscle/liver= 1 µg)

### Liquid chromatography/mass spectrometry (LC/MS) analysis

Negative ion electrospray ionization LC/MS analysis of lipid extracts was performed on a Thermo Scientific (San Jose, CA, USA) Vantage TSQ mass spectrometer with Thermo Accela UPLC operated by Xcalibur software. Lipids were separated on a Restek 150 × 2.1 mm (5 µm particle size) Viva C_4_ column under established conditions. The tetramyristoyl CL internal standard (m/z 1,240, [M-H]^-^) elutes at 13.6 minutes while tetralinoleoyl CL (m/z 1,448, [M-H]^-^) elutes at 14.4 minutes. Complete details of CL analysis by LC/MS are provided elsewhere^[16, 21–24]^_._

### Statistical analysis

Statistical analyses were performed using the Student's *t*-test and data are shown as mean±SEM where *P*≤0.05 is considered significant.

## Results

### Single dose CL-ND toxicity study

**Fig.1:**
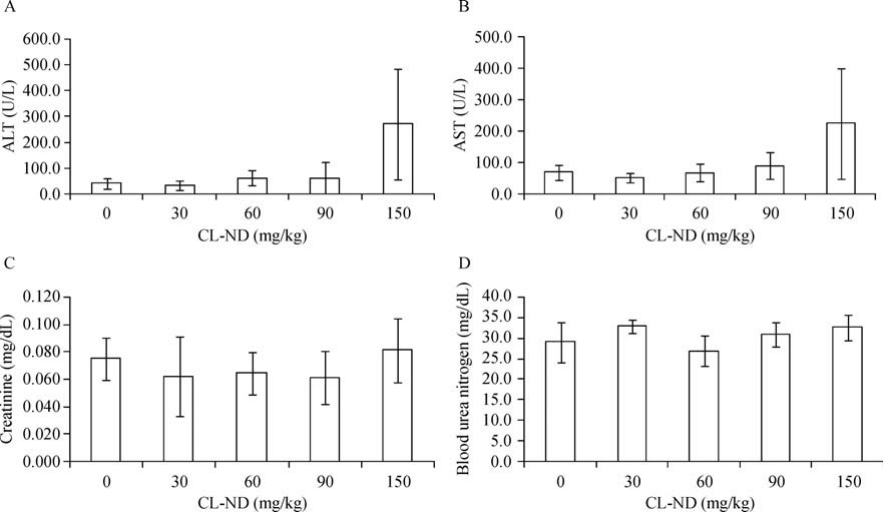
Effect of CL-ND administration on liver and kidney toxicity. WT C57BL/6J mice were injected with escalating doses of CL-ND. Twenty-four hours after CL-ND administration, blood was collected and plasma analyzed for liver enzyme (ALT and AST) activity. Kidney toxicity was evaluated by measuring plasma levels of creatinine, and blood urea nitrogen. Results are reported as mean ±SEM (*n* = 5). CL: cardiolipin; ND: nanodisk.

To identify a safe, nontoxic dose of CL-ND, WT mice were subjected to a single injection protocol. Groups of 5 mice each were injected with 0, 30, 60, 90 and 150 mg/kg CL as CL-ND. After 24 h, plasma was obtained and analyzed for evidence of kidney and liver toxicity (***Fig. 1***). Plasma levels of the liver enzymes, ALT and AST, were within the normal range at all CL concentrations tested. However, at the highest dose administered (150 mg/kg CL), a trend toward increased activity for both enzymes was noted, suggesting a liver toxicity threshold. At the same time, no CL concentration-dependent differences were observed in the levels of creatinine or BUN and all values were within the normal range, consistent with normal kidney function. Based on these findings, a dose of 90 mg/kg CL, in the form of ND, was employed in subsequent studies.

### CL-ND administration to ***taz*** shRNA KD mice

Two approaches to CL-ND administration were employed. In the first, a single weekly dose of 90 mg/kg was injected IP, while the second approach employed 5 daily injections of 18 mg/kg CL-ND IP. Groups of *taz* shRNA KD mice received a) daily or b) weekly injections of CL-ND for 10 weeks while control *taz* shRNA KD mice received weekly injections of PBS. WT mice were administered either PBS or CL-ND. Over the 10-week treatment period, mice were monitored for activity and outward signs of ill health. No differences in activity between groups were detected by visual comparison, and for the first 8 weeks of the treatment phase, all mice appeared healthy. During the final 2 weeks of treatment, however, three *taz* shRNA KD mice in the 90 mg/kg CL-ND weekly bolus treatment group died, two at week 9 and the third at week 10. Thus, in this treatment group, only 50% of the mice survived the 10-week treatment phase. On the other hand, all mice in the daily injection group (18 mg CL/kg) survived the treatment phase and appeared healthy throughout. Based on this discrepancy, it may be concluded that daily dosing is better tolerated than a single weekly bolus injection.

**Fig.2:**
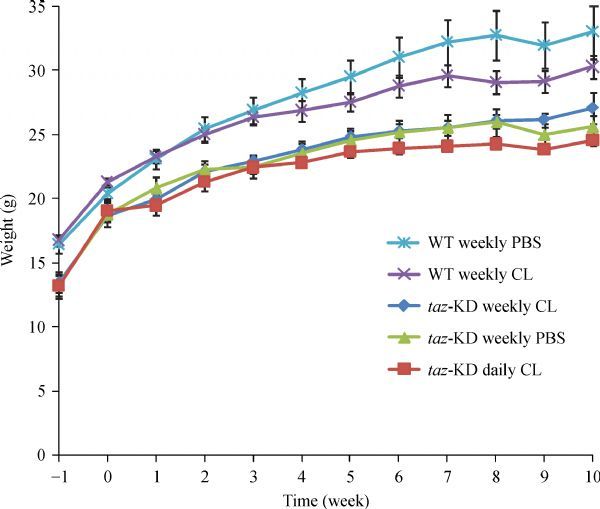
Effect of CL-ND administration on mouse weight gain. Mice were weighed upon arrival (week –1) and every week thereafter until the conclusion of the experiment. Results are reported as mean ±SEM. CL: cardiolipin; KD: knockdown.

From the outset, *taz* shRNA KD mice were smaller and weighed less than control WT C57BL/6J mice and this difference in weight persisted throughout the treatment phase of the experiment (***Fig. 2***). On average, *taz* shRNA KD mice weighed 16% less than their WT littermates (*P*<0.0005), a result that is consistent with the findings of Cole* et al.*^[25]^ Although no mortality was observed in WT C57BL/6J mice administered a 90 mg/kg weekly bolus of CL-ND, a trend toward reduced weight gain over time was observed. In the case of WT mice, the only CL-ND injection group was 90 mg/kg per week. Thus, it remains unclear if a daily injection protocol would have the same effect on weight gain in WT mice.

### CL levels in key tissues

**Fig.3:**
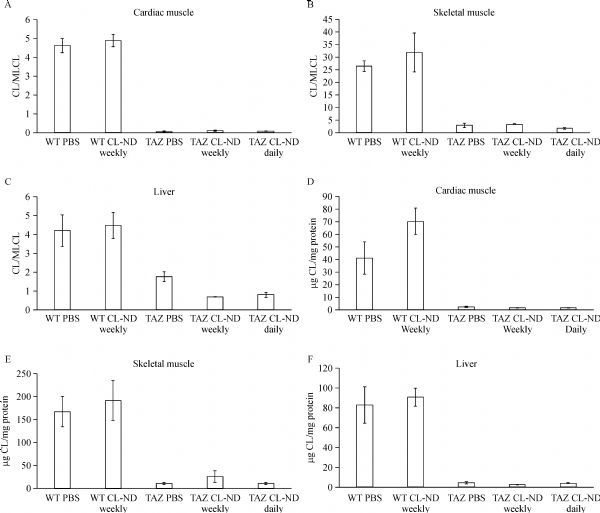
Cardiolipin analysis. At the end of the treatment period, organs were harvested from both *taz* shRNA KD mice (TAZ) and wild type littermates (WT), homogenized, and subject to lipid extraction. The ratio of tetralinoleoyl CL to trilinoleoyl MLCL (CL/MLCL) was quantified by LC/MS (A-C). The absolute amount of tetralinoleoyl CL was quantified relative to a tetramyristoyl CL internal standard and presented as tetralinoleoyl CL relative to protein (D-F). Data are presented as mean ± SEM. CL: cardiolipin; ND: nanodisk; TAZ: taffazin.

After 10 weeks of treatment, mice were sacrificed, tissues harvested, flash frozen and stored at -80ˆC until extraction and analysis by LC/MS. Consistent with known effects of *taz* KD, heart, muscle, and liver tissue of *taz* shRNA KD mice had a significantly lower ratio of CL/MLCL, as compared to the same tissues from WT mice (***Fig. 3A-C***). CL-ND treatment, either as a weekly bolus injection or daily injection, failed to induce a change in the CL/MLCL ratio in tissues from WT or *taz* shRNA KD mice. Absolute levels of tetralinoleoyl CL followed a similar pattern (***Fig. 3D-E***).

## Discussion

ND technology has been exploited for numerous applications including: packaging transmembrane proteins in a native-like membrane environment, solubilization of hydrophobic biomolecules, and as a transport vehicle for contrast agents used in magnetic resonance imaging of atherosclerotic lesions^[15]^. Recently, ND have been shown to be an effective means to solubilize CL^[16]^. Negative stain electron microscopy revealed that tetralinoleoyl CL-ND are discoidal particles with a diameter in the range of 18–31 nm. Thus, stable and water-soluble CL-ND can be generated via a facile one-step formulation process.

Makaryan *et al.*^[13]^ reported that shRNA-mediated KD of *TAZ* in HL60 myeloid progenitor cells leads to induction of apoptosis. Ikon *et al*.^[16]^ confirmed this effect but also found that incubation of these cells with exogenous tetralinoleoyl CL-ND attenuates the apoptotic response. When HL60 cells were incubated with CL-ND harboring trace amounts of a fluorescent CL analog, it was determined that exogenous CL localizes to mitochondria. These results suggest that exogenous tetralinoleoyl CL, solubilized in ND, is taken up by HL60 cells and utilized in lieu of *de novo* synthesized and/or remodeled cardiolipin. The cell culture studies also suggested that CL-ND have the capability to deliver CL to cells *in vivo*, and thus may have therapeutic potential.

The goal of the present study was to extend these *in vitro* findings to an *in vivo *setting, with a view to CL replacement therapy as a treatment strategy for BTHS. Studies were performed using the dox-inducible *taz* shRNA KD mouse^[17]^, which manifests BTHS-associated CL abnormalities within 8 weeks. Following identification of an optimal CL-ND dose, a 10 week study was conducted to assess the effect of CL-ND administration on: (1) the ratio of CL/MLCL and (2) the abundance of CL in key tissues. The results of LC/MS analysis indicate CL-ND administration does not alter the aberrant CL profile of *taz* shRNA KD mice. Given the relatively high dose of CL administered (90 mg/kg per week), it is unlikely that increasing CL-ND concentration, frequency of administration, or duration of treatment will overcome the transport impediment that prevents exogenously administered CL from reaching mitochondria *in vivo*. These results highlight the difficulties inherent in extrapolation of results from cell culture studies to intact animals. Based on the results obtained in this mouse model of BTHS, we conclude that CL-ND administration is not a viable therapy option for treatment of this rare mitochondrial disorder.
